# Neurodevelopmental origins of self‐limiting rolandic epilepsy: Systematic review of MR imaging studies

**DOI:** 10.1002/epi4.12468

**Published:** 2021-03-02

**Authors:** Stuart D. W. Smith, Anna B. Smith, Mark P. Richardson, Deb K. Pal

**Affiliations:** ^1^ Department of Basic & Clinical Neurosciences Institute of Psychiatry, Psychology & Neuroscience King's College London London UK; ^2^ Evelina London Children's Hospital London UK; ^3^ MRC Centre for Neurodevelopmental Disorders King's College London London UK; ^4^ King's College Hospital London UK

## Abstract

**Objective:**

Recent neuroimaging studies have revealed differences in cortical and white matter brain structure in children with self‐limiting rolandic epilepsy (RE). Despite this, reproducibility of the findings has been difficult, and there is no consensus about where and when structural differences are most apparent. We performed a systematic review of quantitative neuroimaging studies in children with RE to explore these questions.

**Methods:**

Using PRISMA guidelines, we used a multilayered search strategy to identify neuroimaging studies in RE. Publications were included if they were quantitative and derived from controlled group studies and passed a quality assessment. Findings of the studies were presented and stratified by duration of epilepsy and age of participants.

**Results:**

We identified six gray matter studies and five white matter studies. Consistent findings were found inside and outside the central sulcus, predominantly within the bilateral frontal and parietal lobes, striatal structures, such as the putamen and white matter, mainly involving the left superior longitudinal fasciculus and connections between the left pre‐ and postcentral gyrus. Stratification of the T1 studies by age found that cortical thickness differences varied between the under and over 10 year olds. Furthermore, the longer the duration of epilepsy, the less likely differences were detected. In white matter studies, there was a reduction in differences with increased age and duration of epilepsy.

**Significance:**

These findings would suggest that the development of regions of the cortex in children with RE is abnormal. These regions are more widespread than the suspected seizure onset zone. Moreover, the findings would suggest that these differences are evidence of neurodevelopmental delay rather than apparent “damage” from the epilepsy.

**Key points**

- Quantitative neuroimaging in children with self‐limiting rolandic epilepsy (RE) can help with understanding the epilepsy mechanism and cooccuring cognitive problems.
- Systematic review was used to identify commonalities between existing studies.
- Differences with healthy controls were seen within and outside the central sulcus. This included white matter structures and subcortical gray matter.
- Stratification of data for age and epilepsy duration demonstrates evidence for a disorder of neurodevelopment.
- Evidence does not support seizures in RE leading to damage.

## 1 INTRODUCTION

Self‐limiting rolandic epilepsy (RE) is a common childhood epilepsy,^1^, ^2^, ^3^ which is often associated with neurocognitive problems. These problems may involve speech,^4^ language,^5^ auditory processing,^6^ reading,^7^ attention,^8^ memory,^9^ motor function,^10^ and visuospatial skills.^11^ These problems are not unique to the epilepsy and can present before, or following, the diagnosis of epilepsy and are mostly not related to seizure frequency.^12^, ^13^ Moreover, there is evidence that similar neurocognitive problems can be apparent in both seizure‐free siblings and parents of children with RE,^14^, ^15^, ^16^, ^17^ with evidence of major genetic influences.^4^, ^18^ Some neurocognitive problems persist after seizure remission,^19^, ^20^, ^21^ and group neuroimaging analyses may reveal subtle abnormalities in brain structure that could explain this.

Routine magnetic resonance imaging (MRI) rarely detects any pathological abnormalities in RE, and incidental findings are comparable to those in normal children.^22^ Indeed, most clinical neuroimaging studies have produced findings of variable quality, such as asymmetrical hippocampi,^23^, ^24^ lateral ventricular dilation,^25^ reduced frontal lobe volume,^26^ and small regions of defective myelination in the frontal and temporal lobes.^23^ However, these studies were suboptimal, used CT scans,^27^ low tesla MRI,^26^ large slice thickness,^23^ lacked controls,^28^ or designed retrospectively.^24^ In contrast, high‐quality research MRI has produced findings that suggest abnormal neurodevelopment, including abnormalities of cortical thickness, volume, and age‐associated thinning.^29^

The primary aim of this review was to identify all of the controlled quantitative MRI studies in children with RE and from this collection identify regions in cortical and subcortical gray and white matter that may have a role to play in neurocognitive dysfunction in this patient group. Our secondary aim was to ascertain how gray and white matter variables change over time. We searched the world literature for cross‐sectional and longitudinal controlled studies with quantitative findings to identify the highest quality evidence.

## 2 METHODS

The systematic review methodology is based on the work of Eggers, Smith, and Altman^30^ and Boland, Cherry, and Dickson.^31^ The strategy of this review was to 1) identify all relevant research, 2) judge the quality of the literature, 3) systematically synthesize the findings of studies of acceptable quality, and 4) make judgments about the research questions.^32^

### 2.1 Search strategy

We used a multilayered search strategy. The first search was performed on 18/04/2017 and was recently updated on 04/05/2020. First, we searched the Prospero and Cochrane databases of systematic reviews using the Medical Subject Headings (MeSH) search term "Rolandic epilepsy". Secondly, we used the MeSH terms "Rolandic epilepsy" and "magnetic resonance imaging" to search three databases (PubMed, Ovid, and Scopus) on 21/04/2017. Thirdly, we conducted a focussed search on the Web of Science database used the MeSH term "Rolandic epilepsy" and the search terms "TOPIC: (rolandic epilepsy) AND TOPIC: (diffusion tensor imaging) AND TOPIC: (DTI) AND TOPIC: (control)" and "TOPIC (rolandic epilepsy) AND TOPIC: (cortical) AND TOPIC: (control)". TOPIC searches titles, abstracts, authors, and keywords while KeyWords Plus^®^ a Web of Science feature augments traditional keyword retrieval. Even though the majority of the neuroimaging literature has been produced over the last decade, there were no date restrictions to the searches. We also searched the bibliographies of all of the identified papers to find additional papers. Finally, we searched for gray (unpublished) literature in archives at King's College London and made correspondence with all major RE research groups.

### 2.2 Inclusion criteria

The following was required for inclusion. Quantitative T1 or diffusion‐weighted imaging (DWI) studies with a control group comparator. The participants were required to have a diagnosis of RE which was either focal or secondary generalized and had to fulfill the criteria set by the International League Against Epilepsy.^33^ No studies were excluded based on diagnostic attributes such as seizure frequency, antiepileptic drug therapy, interictal EEG spike density or location, duration of epilepsy, age of first seizure, or cognitive deficits.

### 2.3 Grouping of studies

The cross‐sectional and longitudinal studies were grouped into the following categories: (i) gray matter: structural analysis of the cortex and subcortical structures from T1‐weighted MRI; (ii) white matter: structural analysis of myelinated brain regions and tracts using DWI.

### 2.4 Quality check

We assessed the selected papers for quality using a modified Newcastle‐Ottawa Quality Assessment Scale for Case‐Control studies^34^ (Table 1). The selection and comparability sections were used with a maximum score of six stars. Studies with less than three stars were excluded from the synthesis of results.

**TABLE 1:** Quality ratings of gray and white matter MRI studies

	Quality rating	Included for analysis	Sample (m:f)		Age, years ± sd		Handedness R/L/Amb/	
			RE	Control	RE	Control	RE	Control
Gray matter studies								
Kohrogi and Mitsudome (1993)	*	No	27 (11:16)	17 (10:7)	9.9 ± 2.7	10.4 ± 2.7	n.r.	n.r.
Kanemura et al (2011)		No	7 (6:1)	11 (8:3)	7	7.8	2/ n.r.	n.r.
Lin et al (2012)	*****	Yes	13 (8:5)	54 (24:30)	10.2 ± 1.4	13.2 ± 3.0	n.r.	n.r.
Overvliet et al (2013)	****	Yes	24 (15:9)	24 (14:10)	11.3 ± 1.9	10.6 ± 1.8	20/3/1	22/2/0
Pardoe et al (Group A)	******	Yes	16 (11:5)	20 (12:8)	9.3 ± 1.6	9.7 ± 1.6	16/0/0	18/2/0
Pardoe et al (Group B)	****	Yes	9 (3:6)	35 (17:18)	15.8 ± 2.3	16.9 ± 4.6	8/1/0	n.r.
Pardoe et al (Group C)	****	Yes	10 (3:7)	20 (8:12)	22.7 ± 2.7	23.7 ± 3.4	9/1/0	n.r.
Garcia‐Ramos et al (2015)	******	Yes	13 (7:6)	24 (11:13)	10.3 ± 1.9	11.3 ± 2.0	n.r.	n.r.
Kim et al (2015)	****	Yes	20 (14:6)	20 (14:6)	7.5 ± 1.5	7.4 ± 1.5	14/2/0	14/2/2/2
Luo et al (2015)	****	Yes	21 (13:8)	20 (10:10)	9.1 ± 1.5	9.3 ± 2.0	21/0/0	20/0/0
Shakeri et al (2017)	**	No	41 (28:13)	38 (25:13)	10.34 ± 1.73	11.18 ± 1.75	n.r	n.r.
Fujiwara et al (2018)	****	Yes	23 (13/10)	32 (15/17)	8.61 ± 2.01	8.31 ± 2.26	21/2/0	32/0/0
Karalok et al 2019	**	No	33 (23/10)	48 (27:21)	8.72 ± 2.44	9.58 ± 2.56	31/2/0	46/2/0
White matter studies								
Besseling et al (2013)	****	Yes	23 (14:9)	23 (12:11)	11.4 ± 2	10.4 ± 1.6	19/3/1	12/2/0
Ciumas et al (2014)	*****	Yes	25 (18:7)	25 (14:11)	9.6 ± 1.9	10 ± 3	20/3/2/	19/5/1
Kim et al (2014)	*****	Yes	19 (11:8)	25 (14;11)	10.7 ± 2.4	10.4 ±.2.7	n.r.	n.r.
Xiao et al (2014)	***	Yes	28 (14:14)	18 (11:7)	9.4 ± 2.3	10.1 ± 1.9	28/0/0	18/0/0
Wu et al (2015)	**	No	32 (15:17)	25 (15/10)	9.8 ± 1.5	10.0 ± 1.5	31/1/0	23/2/0
Cao e al (2017)	**	No	38 (22:16)	20 (13/7)	9.43 ± 1.7	9.67 ± 2.55	n.r.	n.r.
Ostrowski et al	****	Yes	20 (16:4)	14 (7:7)	11.5 ± 1.9	9.8 ± 2.1	2/18/0	11/2/1

Note

Papers with three or more stars were included for analysis.

### 2.5 Extraction of data

In the resulting papers and abstracts, we recorded details from T1 MRI studies of brain regions or structures where the study's authors detected a significant difference in cortical thickness (mm), or volume (mm^3^). This included subcortical structures and their shape. In the DWI studies, we investigated white matter within the whole‐brain, tracts, and U‐shaped fibers, where the authors detected either a significant difference in fractional anisotropy (FA, a scalar value between 0 and 1) or a mean diffusivity (MD, mean of three eigenvalues which form the tensor). We chose FA and MD, as they are complementary measures of white matter integrity. MD is suspected to be a measure of membrane density and structure within axons, which reduces with myelination and axonal packing. FA is regarded as a measure of white matter integrity, and it increases with the myelination of axons and axonal packing.^35^

## 3 RESULTS

**FIGURE 1:**
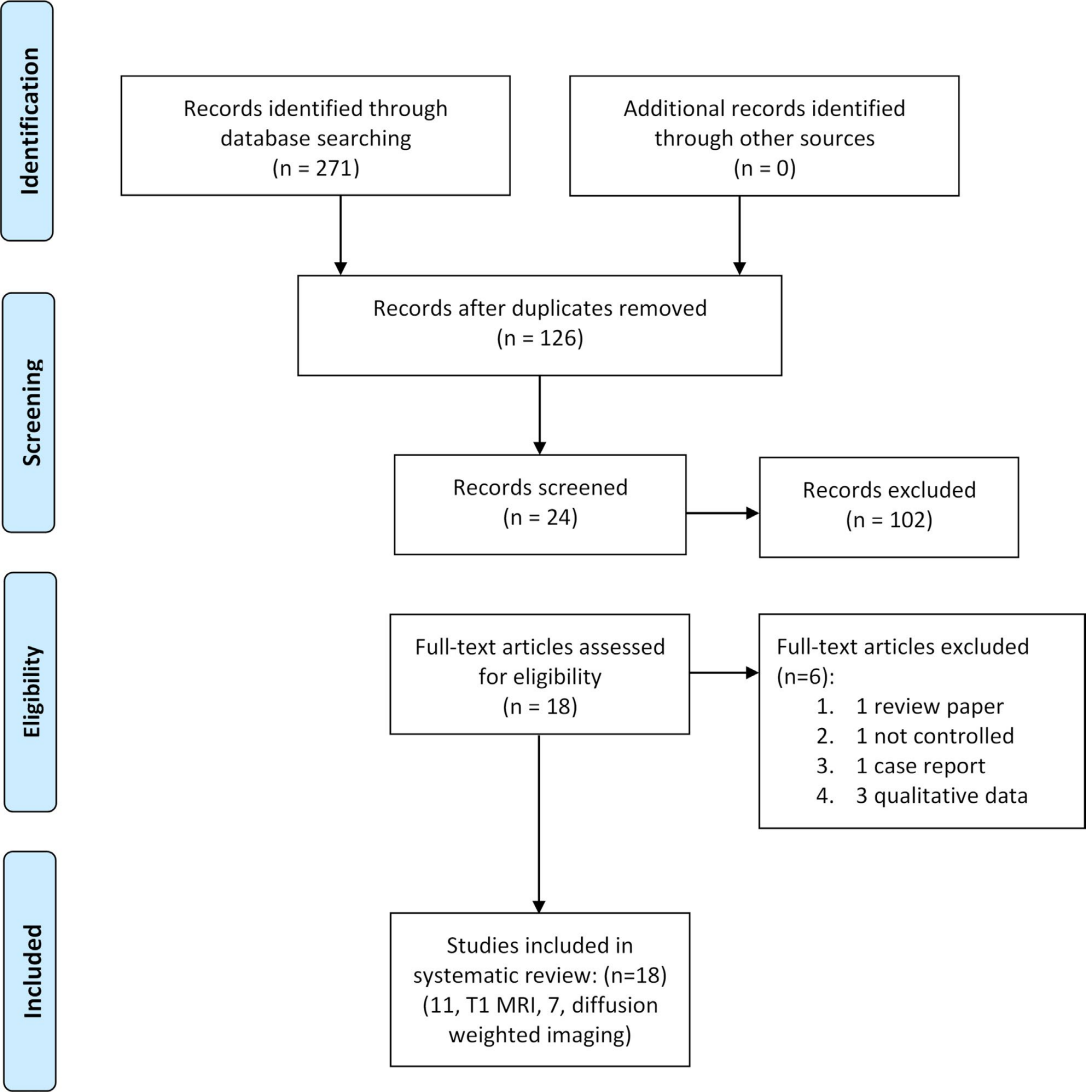
PRISMA flowchart of the systematic review process

We identified 271 papers in the search process: 18 papers remained after exclusion criteria were applied. Eleven publications analyzed structural T1 studies and seven analyzed DWI. The search and screening process is detailed in the PRISMA^36^ flow diagram (Figure 1).

### 3.1 Quality check

#### 3.1.1 Gray matter

Six publications consisting of nine groups were included (Table 1). The range of quality was wide (0‐6 stars), mean 3.27 ± 1.71 stars. Two studies, Garcia‐Ramos^29^ and Group A of the Pardoe study, fulfilled all of the criteria.^37^ Quality was lost in creating a representative sample of children with RE and the reporting on controls selection. Four papers^38^, ^39^, ^40^, ^41^ failed the minimum quality check and were removed.

#### 3.1.2 White matter

Five publications consisting of six groups were included (Table 1). None of the studies obtained a full quality score. The range of quality was 2‐5 stars, mean 3.57 ± 1.27 stars. Quality was lost in using representative cases and underreporting of control recruitment. Two studies^42^, ^43^ scored below average and were excluded.

### 3.2 Search findings

#### 3.2.1 Gray matter, cortical thickness

Three studies reported regions of thinner cortex^29^, ^37^, ^44^ (Pardoe et al, Group B), involving the bilateral middle frontal gyrus and the left superior and inferior temporal gyrus. One study^45^ reported no differences between the groups. The only longitudinal study reported a significant reduction in cortical thinning in children with RE compared to controls at follow‐up. This included regions in the left rostral middle frontal gyrus, insula, bilateral occipital gyrus, postcentral gyrus and right superior frontal gyrus, and precentral gyrus.^29^ Two studies found significantly thicker cortical regions^37^, ^46^ (Pardoe et al, Group A) in the bilateral middle and inferior frontal gyri and right superior frontal gyrus (Table 2).

**TABLE 2:** Table of putamina effect sizes and overall average for each study that reported on putamen/putamina volumes

Study	Left Putamen			Right Putamen			Combined Putamen		
	RE (mm^3^)	Control (mm^3^)	Effect size (*d*)	RE (mm^3^)	Control (mm^3^)	Effect size (*d*)	RE	Control	Effect size (*d*)
Lin et al 2012	n/a	n/a	n/a	n/a	n/a	n/a	11 133 ± 1022	10 441 ± 684	0.97
Garcia‐Ramos et al 2015	6687 ± 142	6246 ± 106	0.86	6499 ± 146	6121 ± 109	0.71	n/a	n/a	n/a
Kim et al 2015	6194.9 ± 547.8	5609.4 ± 687.7	0.97	6156.8 ± 509.5	5545.8 ± 608.6	1.13	n/a	n/a	n/a
Luo et al 2015	59 voxel cluster t statistic : 3.61		1.14	202 voxel cluster t statistic : 4.02		1.27	n/a	n/a	n/a
Average	6388 8 ± 390.7	5956.6 ± 369.5	0.99	6291.6 ± 368.8	5859.5 ± 335	1.04	11 133 ± 1022	10 441 ± 684	0.97

#### 3.2.2 Gray matter, cortical volume

In contrast to cortical thickness, an increase in gray matter volume was seen predominantly in subcortical structures. Six cohorts^29^, ^37^, ^46^, ^47^ showed an increase in gray matter volume in the neocortex, and putamina volumes were larger than controls in four studies with a mean effect size, *Cohen's d = *0.97 (Table 2).^29^, ^46^, ^48^, ^49^ Longitudinal measurement^29^ shows that in children with RE, the uncorrected putamen volume subtly increases over time; the right putamen changed the most. This was counter to controls where putamen volume decreased. Other enlarged subcortical structures included the bilateral amygdalae (volume)^46^ and the left caudate (shape).^48^ Increased cortical volume was detected in parts of the bilateral middle frontal gyrus, but this was only reproducible between the groups in the Pardoe study.^37^

**TABLE 3:** Gray matter studies stratified by group mean age and mean duration of epilepsy

Study	Mean age (years ± SD)	Mean epilepsy duration (months)	Measure	Frontal lobe		Temporal lobe		Parietal lobe		Occipital lobe	
				L	R	L	R	L	R	L	R
Kim et al (2015)	7.5 ± 1.5		Cortical thickness								
		6 ± 0.5	Cortical volume								
Fujiwara et al (2018)	8.61 ± 2.01		Cortical thickness								
		2	Cortical thickness								
Luo et al (2015)	9.1 ± 1.5		Cortical volume								
		13.4 ± 12.9	Cortical volume								
Pardoe et al (2013) (A)	9.3 ± 1.6		Cortical volume								
		<5	Cortical volume								
Pardoe et al (2013) (B)	15.8 ± 2.3		Cortical thickness								
	15.8 ± 2.3		Cortical volume								
Pardoe et al (2013) (C)	22.7 ± 2.7		Cortical volume								
Overvliet et al (2013)	11.3 ± 1.9		Cortical thickness								
		28.8 ± 0.2	Cortical thickness								
Garcia‐Ramos et al (2015)	10.3 ± 1.9^a^		Cortical thickness								
		6.5 ± 3.4	Cortical thickness								
	12.3^a^		Cortical thickness								

Note

Differences include increased (hatched) or decreased (dotted) thickness and volume. Included are brain regions and the laterality of the effect.

^a^ Baseline and longitudinal results from same participants.

#### 3.2.3 White matter

Nearly all studies reported values of mean diffusivity (MD) and fractional anisotropy (FA), except for one,^50^ which omitted MD values. Half of the studies reported tract‐based findings^50^, ^51^, ^52^ one reported on" streamlines"^50^; one partly reported on U‐shaped fibers of the perirolandic regions^53^ and the rest for brain regions.^50^, ^53^, ^54^ Points of agreement were found for both MD and FA measures.

Fractional anisotropy (FA) was reported in all four studies. Differences in whole‐brain FA were reported in 50% of the studies, and all reported a decrease in FA.^50^, ^53^, ^54^ Four studies found regions of reduced FA within tracts with the left longitudinal fasciculus and the anterior thalamic radiation most frequently reported. Only one study specifically studied U‐shaped fibers, and this identified increased FA within the perirolandic regions compared to controls.^53^ Similar features were seen with measures of MD.

Whole‐brain, deep white matter MD was reported in two studies, but only one measured a detectable difference in MD^54^ which was found within the bilateral postcentral gyrus, middle frontal gyrus, cuneus, and left parietal lobe. Differences in tract‐based MD were reported in three studies,^51^, ^52^ and increased MD within the left superior longitudinal fasciculus was a point of congruence. The study which analyzed U‐shaped fibers found increased MD within the perirolandic regions.

In summary, differences in MD and FA can be detected in many different white matter tracts in children with RE, predominantly in the left hemisphere, in particular the superior longitudinal fasciculus and the pre‐ and postcentral gyri.

### 3.3 Data analysis

To simplify cortical data, we sorted by cortical lobe, volume, or thickness measures or both, and stratified by mean age and duration of epilepsy (Table 3). Cortical differences compared with healthy controls were apparent in all studies within a broad age range (7‐22 years).

#### 3.3.1 Age stratification

There appeared to be a difference between cortical thickness in younger children (<10 years) and older children (>10 years). In younger children, thicker cortical regions were more apparent^37^, ^46^, ^47^ (Pardoe et al, Group A), including regions of the bilateral frontal, right temporal, and parietal lobes. In older children, decreased cortical thickness was more apparent^29^, ^37^, ^44^ (Pardoe et al, Group B) seen predominantly in the parietal lobe (3 studies, right‐sided emphasis). In contrast, the bilateral and left frontal (two studies), left temporal (two studies), and left occipital (one study) lobes were all identified to be thicker in older children.

Similarly, increased cortical volumes were more apparent in younger children compared with older. In younger children, the bilateral frontal (two studies), right temporal (one study), and bilateral and left parietal lobes (two studies) showed regions of increased volume. Increased volume was less prevalent in adolescents and adults and included the bilateral and left frontal (two studies), left temporal (one study), and the bilateral parietal lobe (one study). Only one study demonstrated a decrease in volume, and this was in an adolescent group (Pardoe et al, Group B), within the right frontal lobe.

#### 3.3.2 Epilepsy duration

Epilepsy durations of less than one year showed both cortical thickening and thinning. The Garcia‐Ramos *et* al study demonstrated thinner cortex and did not fit the trend of thicker cortex in other studies. A duration of epilepsy over a year also demonstrated mixed results with both evidence of increased and decreased thickness. The Overvliet *et al* study had the longest epilepsy durations of 28.8 ± 0.16 months and demonstrated the least amount of regional differences with controls, only the left temporal and parietal lobes demonstrating increased in cortical thickness.

The sole longitudinal study in this review^29^ demonstrated at baseline, regions of thinner cortex in the bilateral frontal lobe, left temporal and occipital, and the right temporal lobes compared with controls. In the two‐year follow‐up, reduced cortical thinning was seen in the right frontal lobe, left temporal lobe, and bilateral occipital lobes within regions that were previously thin compared with controls.

Overall, significant differences in cortical thickness/volume were seen across the age ranges. In particular, cross‐sectional studies showed increased cortical volume and thickness in younger children (<10 years) whereas in older children (>10 years) thinner cortical regions and smaller volumes are the most apparent. The association of epilepsy duration on brain structure is unclear; however, it appears that children with a duration of epilepsy over 2 years demonstrate fewer brain changes in both cortical thickness and/or volume compared to children with new‐onset RE.

#### 3.3.3 White matter

A simplification of the FA and MD data for tracts derived from TBSS data is represented in Table 4. A common methodology TBSS was used in three studies (four groups).^50^, ^51^, ^52^ These data were stratified by mean age at scan and mean duration of epilepsy. Across all of the studies, the external capsule, fornix, and midbrain white matter structures were similar to healthy controls. Despite this, differences in FA and MD can be found in the cingulum, corpus callosum, corticospinal tract, inferior network, internal capsule, and thalamic radiations and perisylvian networks. However, there is a lack of congruence between the studies except for findings of decreased FA and increased MD in the internal capsule and thalamic radiations and perisylvian networks (left hemisphere emphasis). Most bilateral changes were seen in Group B.^52^ This group had a mean age of 10.03 ± 2.45 years and contained children with only left sided EEG spikes. However, in other studies with similar or older age groups, differences in white matter tracts were scanty.^50^, ^51^

**TABLE 4:** Findings of TBSS in white matter studies stratified by age and epilepsy duration

Study	Mean age (years)	Mean epilepsy duration (months)	Measure	Cingulum		Corpus Callosum		Corticospinal tract		External capsule		Fornix		Inferior network		Internal capsule and thalamic radiations		Midbrain		Perisylvian networks	
				L	R	L	R	L	R	L	R	L	R	L	R	L	R	L	R	L	R
Xiao et al (2014) (Group A)	8.87 ± 2.23	1.26 ± 0.5	MD																		
			FA																		
Xiao et al (2014) (Group B)	10.03 ± 2.45	1.16 ± 0.1	MD																		
			FA																		
Kim et al (2014).	10.7 ± 2.4	3.3 ± 1.5	MD																		
			FA																		
Besseling et al (2013)	11.4 ± 2	3.9 ± 2.1	MD																		
			FA																		

Note

Measurements of FA and/or MD are presented with the distribution of the affected white matter structures. Hatched squares—significant difference. Dotted squares—no significant difference. Top row JHU white matter tract regions some have been grouped together for ease of understanding.

Abbreviations: L, left; R, right.

#### 3.3.4 Age stratification and epilepsy duration

In studies where seizures have been present for one year, there is evidence of multiple, predominantly bilateral, differences in white matter structures.^52^ In groups three years after the onset of seizures, the amount of white matter changes are minimal and only involve the internal capsule and perisylvian networks.^51^ In groups close to four years epilepsy duration, there are zero differences in FA and MD.^50^

Overall, it appears that many large white matter structures are affected in children with RE. These differences, mostly in MD, are predominantly in large white matter tracts that stretch within and between hemispheres. Smaller white matter structures and those in the midbrain appear to be untouched. Differences with healthy controls are seen in studies where the patients are close to the onset of seizures and/or in younger children. In children who have had a longer duration of epilepsy, the differences with controls are minimal or even nonexistent.

## 4 DISCUSSION

There is evidence from the 12 reviewed studies for both gray and white matter differences in children with RE. These changes are diffuse and involve the following: (a) cortical regions, inside and outside the central sulcus, predominantly within the bilateral frontal and parietal lobes; (b) striatal structures, such as the greatly enlarged putamina; (c) extensive white matter differences, mainly involving the left superior longitudinal fasciculus and connections between the left pre‐ and postcentral gyri. Overall, the evidence favors a developmental rather than seizure‐related cause for the observed changes.

### 4.1 Evidence for delayed development

Age‐stratified findings suggest that the difference in RE brains could be the result of abnormal neurodevelopment. A thicker cortex in younger RE children (<10 years) could suggest either a delay to the normal (thinning) process, an overgrowth of cortical tissue during early development, or an underdevelopment of the white matter at the cortical border, which, due to partial volume effect, would make gray matter appear thicker.^55^ In older patients (>10 years), regions of thinning are more apparent. This raises two alternative possibilities: (i) a different neurodevelopmental trajectory, such as speeded up cortical development with overpruning; or (ii) potential damage to the cortex due to repeated interictal discharges or seizures. Four lines of evidence support the neurodevelopmental explanation.

**FIGURE 2:**
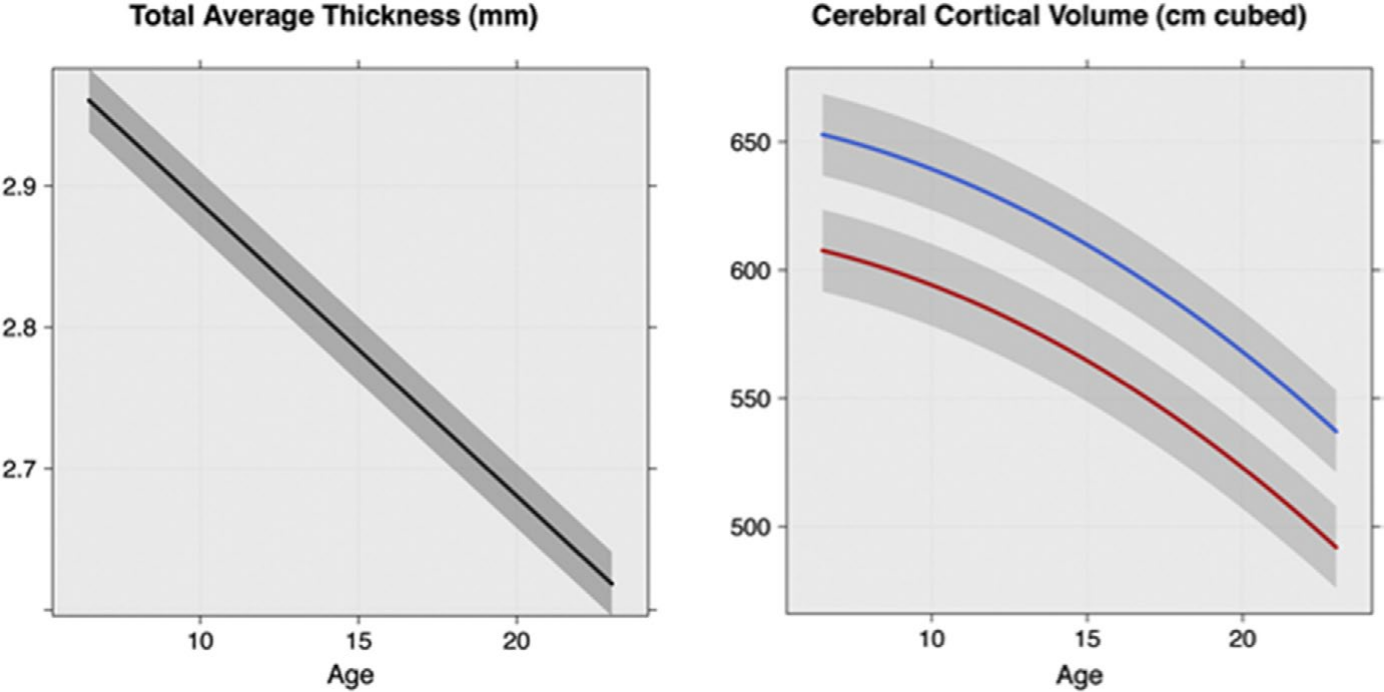
The developmental trajectories of average cortical thickness (in mm) and overall cortical volume (in cm^3^) in healthy children. The cortical volume trajectories are shown in blue for males and in red for females. Extracted from^84^

First, although the sole longitudinal study diverges at baseline from increased cortical thickness found in other studies, it converges with all the other cross‐sectional studies in regard to evidence for delayed cortical thinning. This could be mechanism behind the appearance of thicker cortex in the cross‐sectional studies. Furthermore, in the longitudinal study reduced thinning occurred in regions which were previously thin compared with controls and this may explain thinner cortex in the over 10 years old. All studies have demonstrated that differences in cortical thickness and/or volume in children with RE are patchy and regionalized, the distribution varying with age. Global thinning of the cortex is the predominant process during childhood neurodevelopment from three years up to young adulthood.^56^, ^57^ It is proposed that global cortical thinning predominantly represents a reduction in synaptic density and the resulting loss of dendritic connections within the cortical column.^58^ Figure 2 shows the normal downward trajectories of cortical thickness and volume with age, thinning is linear, and volume reduction is curvilinear; we hypothesize that these trajectories are steeper and regionalized in RE.

Second, the white matter TBSS data indicate that earlier developed tracts such as the optic radiation and external capsule mature normally, whereas the later developing association and projection tracts,^59^, ^60^, ^61^ such as the left superior longitudinal fasciculus (SLF), and the left and right internal capsule and thalamic radiations, appear to contain aberrant regions. In other words, the data indicate a delay to white matter development after a period of normal development.

Third, evidence for similar patterns of altered gray and white matter can be seen in other disorders of neurodevelopment. Increased cortical thickness and/or volume in regions of the frontal lobe have been reported in children with specific language impairment (SLI),^62^ developmental coordination disorder (DCD),^63^ attention‐deficit/hyperactivity disorder (ADHD),^63^ and autism spectrum disorders (ASD).^64^, ^65^ Moreover, increased volume can be seen in the left putamen of children with ASD^66^ and left or right putamen in speech and language disorders^67^ with similar effect sizes to those found in this review. Longitudinal studies have demonstrated possible cortical overgrowth in the frontal lobes in ASD and ADHD during early childhood;^68^ evidence of accelerated thinning in bilateral parietal, occipital, left frontal, and right temporal regions in ASD;^69^ and decelerated maturation of the prefrontal regions in ADHD during childhood.^70^ Neuroimaging studies of white matter have found a decrease in FA and the volume of the SLF in children with dyslexia,^71^ ASD,^72^ and developmental coordination disorder.^73^ Interestingly, in healthy children, increased FA within the SLF is associated with improvement in language and attentional abilities.^74^ While this suggests that maturity of this white matter tract is necessary for these cognitive improvements, there are no longitudinal studies to reinforce these findings.

Fourth, an increased duration of epilepsy did not appear to be associated with an increase in neuroimaging differences, which is counter to a seizures causing damage narrative. Rather, there was evidence to suggest the opposite. When groups with the shortest and longest epilepsy duration were compared, increased thickness and volume of regions within the bilateral frontal and left parietal lobes were apparent in the former group.^37^ These features became apparent close to onset (<5 months) while in contrast, the group with the longest epilepsy duration (28.8 ± 1.8 months) showed only a limited degree of decreased cortical thickness.^44^ Moreover, in the white matter TBSS data, limited differences were detected in groups with a duration of epilepsy over 3.3 years. These findings suggest that the majority of structural cortical development and white matter tracts mature over time, even after many years of epilepsy. Furthermore, it raises the possibility that the white matter differences in new‐onset epilepsy could be due to delayed development before the onset of the epilepsy.

### 4.2 Seizure remission and the need for longitudinal studies

The majority of the studies in this review were cross‐sectional during the active phase of the epilepsy; therefore, we cannot conclude a trajectory of early cortical thickening progressing to late cortical thinning. The only cross‐sectional study with children in seizure remission^37^ (Group C) found the only difference with controls was an increased cortical volume of a small region of the left middle frontal gyrus.^37^ Longitudinal studies partially support this interpretation.

The sole longitudinal study, where 62.5% of participants were in seizure remission at two‐year follow‐up, found changes to sparse patches of predominantly thicker cortex within the regions that were originally thin.^29^ Despite this incongruence between the cross‐sectional and longitudinal studies, they identify changes in cortical thickness over time in the active phase of the epilepsy.

Interestingly in this same study, changes in putamen volume were apparent at follow‐up: In children with RE, there was a subtle increase in the volume of both putamina over two years and a significant difference with controls remained. These volume differences were bilateral, which is in contrast to the cortical data. These findings suggest that the volume of the putamen could be a marker of aberrant brain development.^75^ This is useful as the putamen is a relatively well‐delineated structure within the subcortical white matter suitable for volumetric analysis. We cannot be certain that small group sizes, and publication bias could influence the apparent large putamen effect sizes. Nevertheless, this abnormal development may be due to abnormal growth or more likely, impaired white matter development. Several scientists have proposed that the typical volume reduction in striatal structures requires increased myelination of local axonal projections.^76^, ^77^, ^78^ An alternative explanation is the enlarged putamen could reflect the relationship between a tendency for larger putamen in developing males^78^ compared with females and the increased incidence and prevalence of RE in the male population.^79^ However, this is unlikely as most studies were balanced or controlled for the sex variable.

Overall, it is relatively unknown the transformations in cortical thickness and volume that occur between active epilepsy and seizure remission, nevertheless using the current data there is an intriguing possibility that seizure remission cooccurs with three processes; (i) normalization of the cortex, (ii) the persistence of an enlarged putamen volume, and (iii) subtle small structural cortical differences.

## 5 RECOMMENDATIONS FOR FUTURE STUDIES

The methodological quality of the identified studies was variable. Simple improvements could be made in reporting clinical variables for cases and selection pathways for controls. Including cases at the ages of early or peak seizure onset would be advantageous^79^ but the most informative studies would be longitudinal in design from close to the time of diagnosis until seizure remission. Only a few studies have measured cortical thickness, and this is an area of interest to understand mechanisms. The quality of the magnetic resonance imaging was often poor: Slice thickness in six studies was over 1.2 mm, which can increase the signal‐to‐noise ratio (SNR), and the likelihood of partial volume effect,^80^ introducing error to measurements. In addition, the correct flip angle and time to repetition (TR) are crucial in reducing the SNR. Two sites in the Pardoe *et al* study used a long TR and short TE (1730 /4.38 ms) with a small flip angle (15°), which will produce a proton density image with little T1 weighting. This would introduce poor white gray matter contrast and may have had a detrimental effect on the neuroimaging analysis. In white matter studies, neuroimaging techniques were better designed but the analysis performed had limitations.

One unifying feature of the white matter studies was the use of tract‐based spatial statistics (TBSS).^81^ However, the TBSS technique has problems with the visualization of fine white matter structures as tracts crossing or voxels nearby to the tracts of interest can influence the resulting average FA skeleton.^82^ Furthermore, the FA skeleton reduces complex tracts into simple one voxel sheet with no‐directional information, which reduces the detection of significantly low FA values outside of the skeleton and increases the likelihood of nonwhite matter influencing the FA skeleton.^83^ Finally, the subsequent analysis of the FA skeleton commonly used the John Hopkins University (JHU) template,^84^ which could lead to misinterpretation of the anatomy. Any further understanding of white matter is hindered by the lack of tractographic and longitudinal studies.

Ideally, a meta‐analysis would have been performed on this data. However, due to the range of methodologies in both the gray matter literature and the white matter literature, varying definitions of brain areas and a lack of reproducible findings, a decision was made not to include this form of analysis, although the mean effect size of the putamen was reported. Hopefully, with time, an increase in the number of studies and the availability of detailed data with means and standard deviations will make meta‐analysis a viable option. Finally, we would like to raise the importance of reproducibility in neuroimaging. Currently, there is a crisis in the field, which in part has been demonstrated with this review. To counter this, new strategies are required, for example, the publishing of effect size maps to identify regions of interest and develop hypotheses. In addition, multiple attempts should be made to replicate important findings rather than a casual acceptance of disproved hypotheses.

## CONFLICT OF INTEREST

None of the authors has any conflict of interest to disclose. We confirm that we have read the Journal's position on issues involved in ethical publication and affirm that this report is consistent with those guidelines.

## Supporting information

Supplementary Material
