# Supplementary material for: Neurodevelopmental origins of self‐limiting rolandic epilepsy: Systematic review of MR imaging studies
Source: Epilepsia Open. 2021 Mar 2;6(2):310–22. doi: 10.1002/epi4.12468 (PMC8166787; doi:10.1002/epi4.12468)
Supplement: Supplementary file 1 — Supplementary Material [file EPI4-6-310-s001.pdf]

Supplementary materials

1. Quality Check

Selection

1) Is the case definition adequate?
a) Yes, with independent validation *
b) Yes, e.g. with record linkage or based on self-reports
c) No description

2) Representatives of the cases
a) Consecutive or obviously representative series of cases *
b) Potential for selection biases or not stated

3) Selection of Controls
a) Community controls *
b) Hospital controls
c) No description

4) Definition of controls
a) No history of disease (endpoint) *
b) No description of source

Comparability

1) Comparability of cases and controls based on the design and or analysis
a) Study controls for age *
b) Study controls for gender or handedness *

Supplementary Figure 1: The modified Newcastle-Ottawa score used for quality assessment. Only selection and comparability sections were used. Furthermore, the covariates of interest were age, gender and handedness. The maximum score in this assessment was six points. Criteria with a (*) had to be fulfilled to obtain a star. If there was any uncertainty the point was not awarded.

Grey matter study	Is the case definition adequate?	Representatives of the cases	Selection of controls	Definition of controls	Comparability of cases and controls based on design and analysis	Stars
Kohrogi & Mitsudome (1993)	Not reported	Not reported	Unclear	1 Star: No history of disease	Unclear	1
Kanemura et al (2011)	Not reported.	Unclear, some children had cognitive regression.	Not reported	Hospital controls	No covariates	0
Lin et al (2012)	1 Star: Independent validation. Based on clinical and EEG data	Unclear. Nocturnal generalised tonic-clonic seizures only. Simple partial during waking hours only.	1 Star: First degree cousins	1 Star: No history of disease.	2 Stars: Age and sex as covariates.	5
Overvliet et al (2013)	1 Star: Independent validation. Based on clinical and EEG data	1 Star: Representative series of cases	Unclear	1 Star: No history of disease.	1 Star: Sex as a covariate	4
Pardoe at al (2013)	1 Star: Independent validation. Based on clinical and EEG data	1 Star: Representative series of cases	Only reported clearly in group A	Only reported that group A were healthy.	2 Stars: Age, sex, brain volume and group as covariates.	4 (6 for Group A)
Garcia-Ramos at al (2015).	1 Star: Independent validation. ILAE Classification	1 Star: Required a diagnosis of epilepsy within the last 12 months	1 Star: Healthy first-degree cousin controls matched for age and gender	1 Star: No history of disease	2 Stars: Multivariate analysis of covariance (MANCOVA) with age, gender and intra-cranial volume (ICV) as covariates.	6

Grey matter study	Is the case definition adequate?	Representatives of the cases	Selection of controls	Definition of controls	Comparability of cases and controls based on design and analysis	Stars
Kim et al (2015)	1 Star: Independent validation. ILAE Classification	1 Star: Representative sample from an epilepsy clinic	Unclear	1 Star: No history of disease.	1 Star: Handedness as covariate	4
Luo et al (2015)	1 Star: Independent validation. Based on clinical and EEG data	Excluded patients with attention deficit hyperactivity disorder (ADHD)	Unclear	1 Star: No history of disease.	2 Stars: Age, sex and whole brain volume as covariates	4
Shakeri et al (2017)	1 Star: Independent validation. Based on clinical and EEG data	1 Star representative sample from a paediatric epilepsy clinic	From hospitals	Was not defined	No covariates	2
Fujiwara et al (2018)	1 Star: Independent validation. Based on ILAE classification.	1 Star representative sample from a paediatric epilepsy clinic	Unclear	Typically developing but unclear if free from disease.	2 Stars: Age, sex and whole brain volume as covariates	4
Karalok et al (2019)	1 Star: Independent validation. Based on clinical and EEG data	A Turkish paediatric neurology centre. Unclear location.	Unclear	1 Star: Exclusion of participants with any past or current medical issues including epilepsy and neuropsychiatric disorders or diagnosis of ADHD, a history of febrile convulsion, or a family history of epilepsy.	No covariates	2

White matter studies	Is the case definition adequate?	Representatives of the cases	Selection of controls	Definition of controls	Comparability of cases and controls based on design and analysis	Stars
Besseling et al (2013)	1 Star: Independent validation. Based on clinical and EEG data	Recruited from a specialised epilepsy centre. Excluded children with dyslexia	No description	1 Star: Healthy controls	2 stars: Age and sex as covariates	4
Ciumas et al (2014)	1 Star: Independent validation. Based on clinical and EEG data	1 Star: Representative from a paediatric epilepsy clinic.	Healthy volunteers. No details of recruitment	1 Star: Healthy controls	2 Stars: Age and sex as covariates	5
Kim et al (2014)	1 Star: Independent validation. Based on clinical and EEG data	Recruited from a paediatric epilepsy clinic. Excluded children with developmental disabilities. Included children with bilateral tonic seizures.	1 Star: Community controls. Close friend of patients. Similar age, gender, socio-economic status and educational level	1 Star: Healthy controls	2 stars: Age and sex as covariates	5
Xiao et al (2014)	1 Star: Independent validation. Diagnosed by the International League Against Epilepsy Criteria.	Recruited from West China Hospital of Sichuan University (one patient had myoclonic seizures, four with very high seizure frequencies)	Healthy volunteers. No details of recruitment.	1 Star: Healthy controls.	1 Star: Age as a covariate.	3

White matter studies	Is the case definition adequate?	Representatives of the cases	Selection of controls	Definition of controls	Comparability of cases and controls based on design and analysis	Stars
Wu et al (2015)	1 Star: Independent validation. Diagnosed by the International League Against Epilepsy Criteria.	Recruited from Beijing Childrens Hospital. Patients <7 and >14 years excluded.	No description of how the controls were obtained.	1 Star: Healthy Controls	No evidence	2
Cao et al (2017)	1 Star: Independent validation. Diagnosed by the International League Against Epilepsy Criteria.	Recruited from the pediatric epilepsy outpatient and inpatient department of North Sichuan Medical College, Nanchong, China.	Healthy volunteers: No details of recruitment	1 Star: Healthy controls	No evidence	2
Ostrowski et al (2019)	1 Star: Independent validation. Diagnosed by the International League Against Epilepsy Criteria.	Unclear where the patients were recruited.	Healthy volunteers: No details of recruitment	1 Star: Healthy controls	2 stars: Age and handedness as covariates	4

Supplementary Table 1: Quality check results using the modified Newcastle Ottawa score. The studies can score a maximum of 6 stars. Three stars was required for inclusion in this review.

2. Grey matter studies

Studies	Seizure onset (years)	Mean duration (months)	Number of seizures	AEDs (Y/N)	Polypharmacy	Spike location	Seizure remission (Y/N)
Lin et al (2012)	9.5±1.4	6.5±3.8	n.r.	8/5	0	n.r.	No
Overvliet et al (2013)	7.3±2.2	28±24	n.r.	16/8	5	n.r.	No
Pardoe et al (2013) (A)	n.r.	n.r.	n.r.	10/6	n.r.	n.r.	n.r.
Pardoe et al (B)	n.r.	n.r.	n.r.	3/6	n.r.	n.r.	Quite probable
Pardoe et al (C)	n.r.	n.r.	n.r.	No	n.r.	n.r.	10
Garcia-Ramos et al (2015).	n.r.	6.5±3.4	n.r.	8/5	n.r.	n.r.	5 at follow-up
Kim et al (2015)	6.9±1.7	n.r.	Prior to diagnosis 3.8±1.3	None	None	4/8/8	n.r.
Luo et al (2015)	8.02±1.64	13.41±12.96	n.r.	9/12	0	4/7/10	No
Fujiwara et al (2018)	9.02 ± 1.96	2	2.62±1.96 before enrolment	None	N/A	4/11/6	No

Supplementary Table 2: Group epilepsy characteristics in grey matter studies. AEDs: Antiepileptic drugs. Spike location Right/Left/Bilateral/none. Seizure remission: Greater than one-year seizure free. n.r. not reported.

Study	Structural MRI Protocol. (TE/TR, Flip Angle and Slice thickness)	Magnet Strength (Tesla)	Analysis
Lin et al (2012).	5 / 24 ms, 40°, 1.5 mm	1.5	FSL FIRST
Overvliet et al (2013).	3.8 / 8.3 ms, n.r., 1 mm	3	Freesurfer
Pardoe et al (2013) (Group A)	1.9 / 9 ms, 20°, 2 mm	3	Freesurfer and SPM
Pardoe et al (2013) (Group B)	4.38 / 1730 ms, 15°, 1.6 mm	1.5	Same as group A
Pardoe et al (2013) (Group C)	3.05 / 1730 ms, 15°, 1.6 mm	1.5	Same as group A
Garcia-Ramos et al (2015).	5.0 / 24 ms, 40°, 1.5 mm	1.5	Freesurfer
Kim et al (2015).	4.6 / 9.8 ms, 8°, 1mm	3	Freesurfer
Luo et al (2015).	1.984 / 6.008 ms, 90°, 1 mm	3	SPM and DARTEL
Fujiwara et al (2018)	3.7 / 8 ms, n.r., 1mm	3	Freesurfer

Supplementary Table 3: Structural magnetic resonance imaging methodologies for grey matter studies.

Study	Hypothesis	Baseline thicker regions	Baseline thinner regions	Follow-up thicker regions	Follow-up thinner regions	Grey matter volumetric or shape difference	Disproved null hypothesis
Lin et al (2012).	Compared to controls 1. Investigate neuroanatomic alterations in children with BECTs. 2. Variations in the shape of subcortical structures.	N/A	N/A	N/A	N/A	Putamen volumes larger than controls. Diffuse hypertrophic regions in rostral and caudal putamen. Left caudate expansion in the dorsal and ventral regions.	Nothing to disprove
Overvliet et al (2013).	The goal of the current study is to investigate whether abnormalities in cortical thickness can be found in RE, both within and beyond the sensori-motor cortex. Furthermore, we investigated whether such abnormalities are in the left perisylvian language areas	None	Left hemisphere. Thinner in the supramarginal gyrus and partly covered the bank of the superior temporal sulcus, the superior temporal gyrus and lower post-central gyrus.	N/A	N/A	None reported	Yes, differences were found within and beyond the sensory motor cortex. In addition, these have been found within the left perisylvian language areas.

Study	Hypothesis	Baseline thicker regions	Baseline thinner regions	Follow-up thicker regions	Follow-up thinner regions	Grey matter volumetric or shape difference	Disproved null hypothesis
Pardoe et al (2013) (Group A)	Examine whether any differences observed might progressively vary as a function of age (maturation)	N/A	N/A	N/A	N/A	Increased volume in regions of the bilateral middle frontal gyrus, cingulate and left superior parietal lobe.	If combined with groups B and C, then differences are apparent.
Pardoe et al (2013) (Group B)	Same as group A	Bilateral middle and inferior frontal gyri and supramarginal gyrus. Left insular cortex. Scattered regions in the parietal lobes.	Part of the right central sulcus	N/A	N/A	Increased volume in parts of the bilateral middle frontal gyrus	If combined with groups A and C, then differences are apparent.
Pardoe et al (2013) (Group C)	Same as group A	No significant differences	No significant differences	N/A	N/A	Increased volume in part of the left middle frontal gyrus	If combined with groups A and B, then differences are apparent.

Study	Hypothesis	Baseline thicker regions	Baseline thinner regions	Follow-up thicker regions	Follow-up thinner regions	Grey matter volumetric or shape difference	Disproved null hypothesis
Garcia-Ramos et al (2015).	Investigating differences in cortical thickness and putaminal volume.	None	Bilateral middle frontal gyrus (rostral), left inferior temporal gyrus, left lateral occipital gyrus and right cuneus.	Left rostral middle frontal gyrus, insula, bilateral occipital gyrus, post-central gyrus and right superior frontal gyrus and pre-central gyrus	Left isthmus of the cingulate.	Increased putamen volumes at baseline and follow-up. Putamen increased in volume over time and this was most pronounced in the right putamina.	Yes, differences were found.
Kim et al (2015).	Case-control study to identify abnormal cortical structures.	Right superior frontal gyrus, right superior and middle temporal gyri and precuneus. Left	Nothing reported	N/A	N/A	Larger putamen and amygdala in BECTs.	Unclear

Study	Hypothesis	Baseline thicker regions	Baseline thinner regions	Follow-up thicker regions	Follow-up thinner regions	Grey matter volumetric or shape difference	Disproved null hypothesis
		orbito-frontal gyrus, pars orbitalis gyrus and precentral gyrus.					
Luo et al (2015).	We hypothesise that the regions with significant changes in GM volume may be involved in functional connectivity abnormalities.	N/A	N/A	N/A	N/A	Increased grey matter volume in the bilateral putamen, and para-central lobule. Right interior insula/frontal operculum, right supplementary motor area (SMA), right inferior temporal gyrus and left cerebellum.	N/A

Study	Hypothesis	Baseline thicker regions	Baseline thinner regions	Follow-up thicker regions	Follow-up thinner regions	Grey matter volumetric or shape difference	Disproved null hypothesis
Fujiwara et al (2018)	We hypothesized that children with BECTS will exhibit atypical relationships between cortical thickness and neuropsychological function. Finally, we hypothesized that regional cortical thickness would vary with centrotemporal spike frequency.	No	No			No	Hypotheses were not well defined. A difference in cortical thickness was found for a sub-group of participants with predominant right-CTS and right spikes greater than 10/minute within the right pars opercularis. A relationship was also found between processing speed index and cortical thickness in the Areas within the right precentral, superior parietal, caudal middle frontal, rostral middle frontal and pars opercularis and left caudal middle frontal and precentral regions

Table 4: Hypotheses and findings for included grey matter studies. Included are cortical thickness and volume findings at baseline or follow-up.

3. White matter

Studies	Age of seizure onset (years)	Duration of epilepsy (months)	Seizure frequency (per year)	AEDs (YES/NO)	Polypharmacy	Spike location	Seizure remission (YES/NO)
Besseling et al (2013)	7.5±2.1	46.8±24.08	n.r.	13/10	4	n.r.	n.r.
Ciumas et al (2014)	8.38±2.14	20.7±20	8.1	10/18	2	2/14/9/0	n.r.
Kim et al (2014)	n.r.	36.39±18	n.r.	14/5	0	4/6/9/0	Three participants were likely in remission
Xiao et al (2014) (Group A)	8.15±2.2	n.r.	At least 2 seizures in the past year	10/5	2	15/0/0/0	No
Xiao et al (2014) (Group B)	8.87±2.35	n.r	At least 2 seizures in the past year	10/3	1	0/13/0/0	No
Ostrowski et al (2019)	8.74 ± 1.46	n.r.	n.r.	7/14	1	Not reported but data is available upon request	Seven participants were likely in remission.

Supplementary Table 5: Group epilepsy characteristics for white matter studies. AEDs: Anti-epileptic drugs. Spike location: Right/Left/Bilateral/None. n.r. not reported.

Study	Structural MRI Protocol (TE/TR, Flip angle, slice thickness)	DWI Protocol (TE/TR, Gradient directions, DWI B-values, Number of B0 images)	Magnet Strength (Tesla)	Methodology for analysing diffusion weighted images.
Besseling et al (2013)	3.8/8.3 ms, n.r., 1 mm.	72/6600 ms, 66, 1200 s/mm², 1	3T	MRTrix and Tract based spatial statistics
Ciumas et al (2014)	3.5/2400 ms, 8°, 1.6 mm.	86/6900 ms, 48, 10,000 s/mm², 4	1.5 T	Voxel based analysis
Kim et al (2014).	4.19 /1160 ms, 15°, 1.2 mm	Not reported, 15, 1000 s/mm², n.r.	3T	Tract based spatial statistics
Xiao et al (2014)	n.r.	93/6800 ms, 20, 1000s/mm², n.r.	3T	Tract based spatial statistics
Ostrowski et al (2019)	1.74/2530 ms, 7°, 1 mm	82/8080 ms, 64 ,2000 s/mm²,	3T	FSLs DTIFIT

Supplementary Table 6: Magnetic resonance imaging methodologies for white matter studies. n.r. not reported.

Study	Hypothesis	RE group MD (Regions or tracts)	RE group FA (Regions or tracts)	Disproved null hypothesis
Besseling et al (2013)	In children with RE white matter tracts connecting to rolandic regions may be compromised.	Not reported	Tractography: Lower FA in streamlines between the bilateral pre and post central gyri and the, ipsilateral insula and superior temporal gyrus. Bilateral pre-central gyri and the superior frontal cortex and the par opercularis. Left precentral gyrus and caudal mid frontal cortex and pars opercularis. Bi-lateral post-central gyri and supramarginal gyri. Local connections between bilateral pre- and post-central gyri. TBSS: No significant difference in FA.	Unclear. The team do not define what compromised means.
Ciumas et al (2014)	In children with RE, the age dependence of the epilepsy and cognitive abnormality suggest the possibility of altered maturation of white matter.	Higher MD, left, post-central gyrus, cuneus, middle frontal gyrus and inferior parietal lobe. Right post-central gyrus, medial frontal gyrus, cuneus (two clusters).	Lower FA in the left precentral and post central gyrus.	Unclear. Measurements of FA and MD appeared to be affected by the duration of the epilepsy.

Study	Hypothesis	RE group MD (Regions or tracts)	RE group FA (Regions or tracts)	Disproved null hypothesis
Kim et al (2014).	Alterations in brain in white matter microstructure in certain brain areas would correlate with specific cognitive impairments in RE.	Higher MD in the left superior longitudinal fasiculus (three clusters), rentrolenticular part of internal capsule, posterior thalamic radiation and sagittal striatum.	No significant difference in FA.	Yes, measurements of white matter changes (increased axial diffusivity) in the left hemisphere correlates with lower verbal IQ.
Xiao et al (2014) (Group A)	White matter integrity is compromised in children with active BECTs those with epileptic foci on the dominant hemisphere for language were more vulnerable to damage from seizures.	Increase in MD in the right inferior fronto-occipital fasciculus, anterior thalamic radiation and left superior longitudinal fasciculus and cingulate gyrus.	Decreased FA in the left superior longitudinal fasciculus, cortical spinal tract, cingulum, anterior thalamic radiation and inferior fronto-occipital fasciculus.	Unclear, white matter changes are seen in children with right sided (non-dominant hemisphere) spikes however it is unknown whether this equates to damage.
Xiao et al (2014) (Group B)	White matter integrity is compromised in children with active RE and those with epileptic foci on the dominant hemisphere for language were more vulnerable to damage from seizures.	Increased MD in body and splenium of the corpus callosum, bilateral superior longitudinal fasiculus, cingulate gyrus and corticospinal tract.	Bilateral reduced FA found in body, splenium of the corpus callosum, forceps minor and major, superior longitudinal fasciculus, cingulate gyrus, anterior thalamic radiation. Right inferior fronto-occipital fasciculus and cortical spinal tract.	Unclear, greater white matter changes are seen in children with Lt sided spikes and RE. However, differences in white matter FA and MD do not equate to damage.

Study	Hypothesis	RE group MD (Regions or tracts)	RE group FA (Regions or tracts)	Disproved null hypothesis
Ostrowski et al (2019)	To test the relationship between focal white matter organization and disease in BECTS, we evaluated a priori a region of interest (ROI) corresponding to the superficial region of u-fibres underlying the seizure onset zone in children with BECTS compared with healthy control (HC) subjects. To verify the spatial specificity of these features, we evaluated superficial white matter ROIs outside of the seizure onset zone and deep white matter properties in these children.	Increased in peri-rolandic regions	Increased in peri-rolandic regions<br>FA reduced in deep white matter in all four lobes.	Unclear: Increased MD and FA is unusual but may be a unique property of developing U shaped fibres. Similarly, this study found reduced FA in deep brain white matter but no increase in MD compared to controls.

Supplementary Table 7: Hypotheses and findings for included white matter studies. Included are differences in MD and FA compared to a control group.
